# Editorial: Minimal intervention dentistry for dental caries management

**DOI:** 10.3389/froh.2025.1565605

**Published:** 2025-02-28

**Authors:** Ollie Yiru Yu, Piyaphong Panpisut, Aylin Baysan, Chun-Hung Chu

**Affiliations:** ^1^ Faculty of Dentistry, The University of Hong Kong, Hong Kong, Hong Kong SAR, China; ^2^ Faculty of Dentistry, Thammasat University, Pathumthani, Thailand; ^3^ School of Medicine and Dentistry, Queen Mary University of London, London, United Kingdom

**Keywords:** dental caries, minimally invasive dentistry, minimal intervention dentistry, cariology, remineralization, demineralization

**Editorial on the Research Topic**
Minimal intervention dentistry for dental caries management

Dental caries, a widespread oral disease, significantly impacts the population's health. This disease can occur at any life stage and affects nearly all adults. Untreated dental caries is the most common global condition, with 2.4 billion adults and 514 million children affected worldwide (1). This disease manifests as the localized destruction of dental hard tissues due to the production of organic acid from carbohydrate fermentation by cariogenic bacteria (2). This process can damage both the coronal dental hard tissue and exposed, vulnerable root surfaces (3), leading to potential pain, infection, apical periodontitis, or abscess formation if left untreated.

Our current understanding of dental caries as a non-communicable, chronic disease influenced by behavior, rather than as an infectious disease, has resulted in a shift in the philosophy of caries prevention and management (4). The traditional clinical approach, which addresses signs and symptoms associated with the disease in its advanced stages and focuses only on restorative therapy, is no longer sufficient as this approach fails to address etiological factors of dental caries. Caries prevention or control cannot be achieved only with restorative procedures. The success of restorative, or surgical management heavily depends on the longevity of dental restorations. However, all types of dental restorations have a limited lifespan, even with advancements in material science in restorative and adhesive dentistry. Repeated re-restorations due to dislodgement and replacement of dental restorations lead to further destruction of dental hard tissue and, ultimately, tooth loss. This has led to a shift in contemporary caries management from the traditional surgical approach to a new concept that focuses on controlling etiological and risk factors (4).

Since 2002, the World Dental Federation (FDI) has endorsed the use of Minimal Intervention Dentistry (MID) for managing dental caries (5). MID aims to preserve dental structure and pulpal vitality, thus extending the lifespan of teeth. The primary goal is to enable disease healing through improved oral health. It emphasizes nonrestorative intervention to inhibit mineral loss at all caries stages, incorporating early caries detection, risk assessment, remineralization of demineralized enamel and dentine, optimal preventive measures, minimally invasive operative interventions, and restoration repair rather than replacement.

The research topic “Frontiers in Oral Health: Minimal Intervention Dentistry for Dental Caries Management” comprises four studies that incorporate elements of the MID concept. Conducted by research teams from Argentina, Germany, Thailand, and the United Kingdom, these studies demonstrate the global acceptance and research interest in the MID concept. The four published studies, comprising three laboratory-based studies and one randomized controlled clinical trial, are all original studies.

Two of the laboratory-based studies explored strategies to enhance the remineralization of demineralized enamel and dentine. The topical application of remineralizing agents is an efficient, cost-effective strategy to promote the remineralization of carious dental hard tissue, making the development and evaluation of novel topical anti-caries agents a popular research direction.

Lanthanide salts like Cerium Chloride (CeCl_3_) show promising potential in managing dental erosion and caries, as observed in *in vitro* studies. A critical consideration for their clinical application is whether lanthanides can penetrate the salivary pellicle, a protein film covering the enamel surface, to interact with the dental hard tissue surface. To address this, Kopp et al. investigated the impact of Cerium Nitrate Hexahydrate [Ce(NO_3_)_3_] and Samarium Nitrate Hexahydrate [Sm(NO_3_)_3_] solutions on human enamel, both with and without a salivary pellicle. The authors discovered that applying these solutions on polished enamel led to superficial accumulation and penetration of Ce and Sm into the enamel surface, altering the enamel's elemental composition, regardless of the presence of a pellicle. This finding enhances our understanding of using Ce(NO_3_)_3_ and Sm(NO_3_)_3_ in managing dental caries as a MID strategy.

In another study, Molina et al. developed a novel solution of silver nanoclusters (AgNCls) synthesized in polymethacrylic acid (PMAA) for caries control through topical application. These authors explored the biological properties of this innovative topical anti-caries agent for arresting dentin caries based on AgNCls synthesized in PMAA. Their results indicated that AgNCls/PMAA exhibited chemical stability, acceptable cytotoxicity, and a potential antibacterial effect against strains associated with caries lesions, even at very low silver concentrations. This study underscored the anti-caries potential of AgNCls/PMAA as a MID strategy for dental caries prevention.

This research topic also features a laboratory-based study and a randomized controlled clinical trial focusing on minimally invasive operative interventions. When dental caries evolves into an advanced stage, leading to cavity formation, minimally invasive restorative treatment is the recommended course of action. This treatment aligns with the MID philosophy, prioritizing the preservation of healthy dental hard tissues and the remineralization of affected carious tissue post-caries removal, thereby extending the lifespan of the natural tooth.

Kitsahawong et al. conducted a two-year clinical trial to compare the long-term status of dental restorations following chemo-mechanical caries removal (CMCR) with those after conventional drilling. Their findings indicate that CMCR is as effective as conventional drilling for complete caries removal and restoration success at 24 months in primary teeth. Even though CMCR necessitates long chair time, this technique results in less treatment-related discomfort, suggesting that CMCR, as an MID approach, could be a promising alternative to conventional “drilling and filling”.

Advancements in material science for restorative dentistry have enabled the implementation of minimally invasive restorative treatment. Alkhouri et al. developed a novel restorative material, Renewal MI, designed to promote the remineralization of dental hard tissues beneath the restoration by interacting with the inorganic and organic components of human dentine. Renewal MI can penetrate and form extended resin tags in dentinal tubules, effectively replacing water in the tubules and limiting any enzyme-catalysed hydrolysis of dentine. The study identifies the components in Renewal MI that encourage tag formation in affected dentine. The authors concluded that a combination of polylysine (PLS), monocalcium phosphate (MCP), 4-methacryloyloxyethyl trimellitate anhydride (4META), and polypropylene glycol dimethacrylate (PPGDMA), along with a low powder-to-liquid ratio, enhances Renewal MI tag formation in demineralized dentine.

In conclusion, this research topic offers original research papers that contribute to the advancement of minimal intervention dentistry research and practice. Both clinicians and researchers should find this research topic a valuable resource for implementing minimal intervention dentistry.
